# Imaging Evaluation for Jaw Deformities: Diagnostic Workup and Pre-Treatment Imaging Checklist for Orthognathic Surgery

**DOI:** 10.3390/diagnostics16020367

**Published:** 2026-01-22

**Authors:** Hiroki Tsurushima, Masafumi Oda, Kaori Kometani-Gunjikake, Tomohiko Shirakawa, Shinobu Matsumoto-Takeda, Nao Wakasugi-Sato, Shun Nishimura, Kazuya Haraguchi, Susumu Nishina, Tatsuo Kawamoto, Manabu Habu, Izumi Yoshioka, Toshiaki Arimatsu, Yasuhiro Morimoto

**Affiliations:** 1 Division of Oral Medicine, Department of Physical Function, Kyushu Dental University, Kitakyushu 803-8580, Japan; r17tsurushima@fa.kyu-dent.ac.jp (H.T.);; 2 Division of Oral and Maxillofacial Radiology, Kyushu Dental University, Kitakyushu 803-8580, Japan; 3 Division of Orofacial Functions and Orthodontics, Kyushu Dental University, Kitakyushu 803-8580, Japan; k-kaori@kyu-dent.ac.jp (K.K.-G.); r16shirakawa@fa.kyu-dent.ac.jp (T.S.); r15kawamoto@fa.kyu-dent.ac.jp (T.K.); 4 Division of Maxillofacial Oral Surgery, Kyushu Dental University, Kitakyushu 803-8580, Japanh-manabu@kyu-dent.ac.jp (M.H.); 5 Arimatsu Orthodontic Dental Office, Kitakyushu 806-0021, Japan

**Keywords:** jaw abnormalities, computed tomography, X-ray, magnetic resonance imaging, cone-beam computed tomography, panoramic radiography, cephalometry

## Abstract

In addition to standardized lateral cephalometric radiographs, comprehensive assessment using dental cone-beam computed tomography (CBCT) and CT has become commonplace in the diagnosis and treatment of jaw deformities. Simulation based on cephalometric and CT data is particularly useful in the management of jaw deformities, both for evaluation and prognostic prediction. As such imaging examinations cover a wide anatomical region, it is not uncommon for various incidental pathologies to be discovered. This review emphasizes the necessity of evaluating the entire imaged area in addition to the chief complaint. Furthermore, it outlines the essential anatomical structures that should be assessed during diagnostic imaging performed prior to representative surgical procedures for jaw deformities (e.g., sagittal split ramus osteotomy and Le Fort I osteotomy). This review paper is descriptive in nature, incorporating our facility’s empirical aspects, and presents representative cases in a narrative format; it is not a systematic review. In other word, as the evidence-based literature does not cover all aspects of pretreatment evaluation, these criteria are based on the past experience of the authors.

## 1. Introduction

Jaw deformities are defined as “morphological and occlusal abnormalities of the maxillofacial region caused by discrepancies in the size, shape, or position of the maxilla and/or mandible and disharmonies in the maxilla-mandibular relationship” [1]. The problems caused by these deformities can be broadly categorized into four types: facial appearance abnormalities, occlusal discrepancies, functional impairments (including issues in mastication, mandibular movement, muscular function, swallowing, perioral muscle function, and speech), and psychosocial challenges [1]. The objective of treatment is to address these issues and improve the patient’s quality of life. Accurate imaging evaluation is indispensable to ensure the safe and appropriate management of jaw deformities. This review discusses the imaging assessments that should be performed before treatment, focusing on the essential role of current imaging technologies in jaw deformity surgery.

## 2. Materials and Methods

This review paper is not a systematic review; rather, it is descriptive in nature, incorporating our facility’s empirical aspects, and presents representative cases in a narrative format. Therefore, the selection of the literature and case studies for this paper was conducted as follows. Based on general textbooks, our organization’s experience, and our own clinical observations, we selected the relevant literature and case studies by reviewing documents and identifying keywords corresponding to document titles.

### 2.1. Objectives of Preoperative Diagnosis for Jaw Deformities

The purposes of preoperative diagnosis can be broadly classified into three categories:

1. To obtain an accurate assessment of the current condition of the jaw deformity, including the assessment of growth potential, prior to the preoperative evaluation;
2. To determine an appropriate treatment plan for correction;
3. To identify potential complications that may interfere with the planned procedures.

The specific evaluation items can vary depending on the type of treatment selected but generally comprise several components. After the objective evaluation of the physical characteristics (height, weight, and facial morphology) on standardized facial photographs, the condition of the oral cavity and the relationship of the dental arch and the cranial base are assessed using intraoral photographs and dental models. Imaging studies are conducted for the evaluation of malocclusion and its causes, skeletal growth, and the condition of the temporomandibular joint (TMJ) (Table 1) [1]. Jaw movement analysis and masticatory efficiency tests are performed to assess orofacial function before treatment. In some cases, a general systemic evaluation including chest X-rays is also conducted.

### 2.2. Overview of Preoperative Imaging Diagnosis for Jaw Deformities

Jaw deformities can be categorized as growth disturbances, congenital abnormalities, and acquired types. When planning treatment for jaw deformities, it is essential to clarify the state and causes of malocclusion. The relationships between the imaging modalities used to evaluate malocclusion and the conditions and causes of malocclusion described in authoritative textbooks are summarized in Table 1 [2]. Among these, the most important imaging evaluation is for morphological abnormalities of the jawbone, seeking to determine whether the cause of malocclusion is dental or skeletal in nature. In addition, imaging enables the assessment of other relevant factors, such as the number and shape of the teeth and any eruption anomalies; dental and periodontal diseases; soft tissue abnormalities; and jawbone lesions. It is also necessary to identify any factors that might interfere with orthodontic or surgical treatment. Appropriate imaging techniques must be used to evaluate these conditions.

Other aspects of malocclusion are better evaluated through clinical tools such as intraoral photographs, dental models, oral function tests, and genetic testing.

Table 1 lists the imaging modalities used for the diagnosis of malocclusion and its causes. According to the “Guidelines for the Diagnosis and Treatment of Jaw Deformities” of the Japanese Society of Oral and Maxillofacial Surgeons, the following imaging modalities are strongly recommended at the S level: panoramic radiographs, four-segment TMJ panoramic views, frontal and lateral cephalograms, computed tomography (CT), and magnetic resonance imaging (MRI) [3]. At the A level, carpal radiographs (hand–wrist radiographs) are recommended to assess the individual’s growth and development.

A critical concept when performing imaging is balancing the biological effects (risks) of radiation exposure against the clinical utility (benefits) of the information obtained. The use of radiation is justified only when the benefit outweighs the risk. Furthermore, even when justified, medical radiation use requires the adoption of optimization measures to reduce the exposure levels as much as possible. In particular, the radiation dose must be minimized and as low as reasonably achievable (ARALA principle). Therefore, all imaging procedures must adhere to the principles of justification and optimization. According to the 2009 guidelines of the European Society of Maxillofacial Radiology, CBCT should only be used when conventional imaging methods with lower radiation exposure, such as panoramic radiographs, are deemed insufficient [4]. When performing oral surgical procedures in addition to orthodontic approaches, primarily for jaw deformities, the evaluation of tumorous or inflammatory lesions occurring in the jawbone and soft tissues is necessary, and CT is used.

### 2.3. Diseases That Take Priority over the Chief Complaint in Imaging Evaluation for Jaw Deformities

Preoperative imaging for jaw deformities commonly captures anatomical areas beyond the immediate region of concern. Therefore, clinicians must not overlook conditions that require more urgent intervention than the deformity itself. It is important to avoid becoming overly focused on the jaw deformity and neglecting other significant findings. This section introduces the conditions frequently encountered in various imaging modalities.

**Figure 1:**
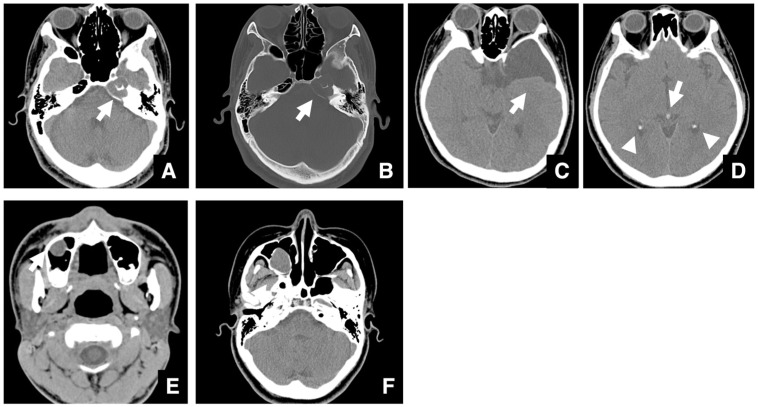
Representative multidetector (MD) row CT images (slice thickness, 3.0 mm) of incidental findings detected during evaluation of jaw deformities. (**A**) Soft tissue and (**B**) bone window images of a patient with a brain tumor (arrow) located in the left petrous region. (**C**) Soft tissue CT image of a patient with an arachnoid cyst (arrow) located anterolaterally on the left. (**D**) Soft tissue CT image of a patient shows calcification of the pineal gland (arrow) and choroid plexus (arrowheads). Soft tissue CT image of a patient with a retention cyst (arrow) in the maxillary sinus. (**E**) The lesion shows lower density than muscle and is not surrounded by bone. Soft tissue CT image of a patient with a dentigerous cyst (arrow) expanding into the maxillary sinus. (**F**) The lesion is surrounded by a thin layer of bone. Permission has been obtained to publish all figures.

Imaging assessment for jaw deformities generally covers a wide area, from the vertex of the skull to the hyoid bone. Although rare, brain tumors may be detected incidentally—for example, as a soft tissue mass in the left petrous portion of the temporal bone with surrounding bone destruction (Figure 1A,B). Even when asymptomatic, referral to an appropriate specialist is warranted. Arachnoid cysts are a relatively common incidental finding in the brain (Figure 1C). It has been reported that arachnoid cysts are present in approximately 1% of patients with jaw deformities [5]. These lesions may be asymptomatic but can cause complications if ruptured due to trauma. When detected, the patient should be informed and referred to the appropriate medical department.

When calcifications are observed within the brain, the presence of a mass must be ruled out. If no mass is present, the calcifications are usually physiological, such as pineal gland or choroid plexus calcifications (Figure 1D), vascular calcifications, or falx cerebri calcifications. Pineal and choroid plexus calcifications are seen in 50–70% of individuals, depending on age [6]. Other frequent calcifications include those of the globus pallidus, cerebral arteries, cerebellar dentate nucleus, and petroclinoid ligament. Chiari malformation, abnormalities of the sella turcica, and other skull base abnormalities can also be detected in imaging studies.

Intraosseous jaw lesions and maxillary sinus masses must also be evaluated. Retention cysts are relatively common in the maxillary sinus, being present in about 20% of cases [5]. These cysts show a low internal density and are not entirely surrounded by bone (Figure 1E). In contrast, dentigerous cysts extending into the sinus are surrounded by a thin layer of bone, suggesting that the mass originated in the jaw (Figure 1F).

**Figure 2:**
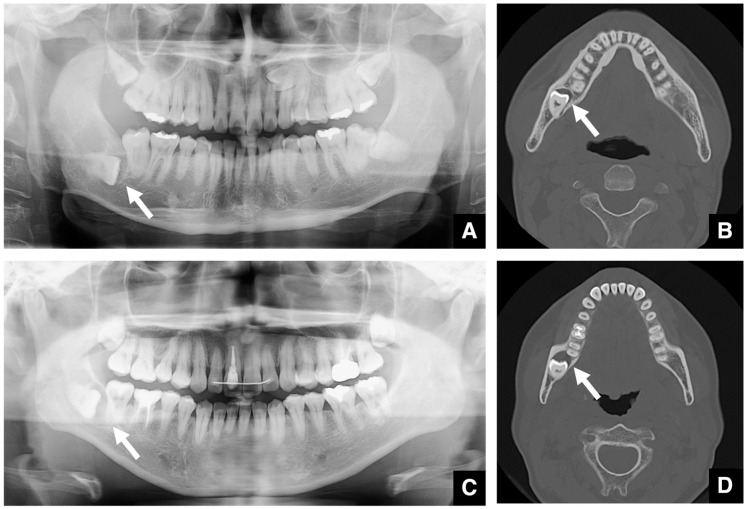
Comparison of dentigerous cyst and normal dental follicle. (**A**) Panoramic radiograph and (**B**) bone window MDCT image (slice thickness, 3.0 mm) of a patient with a normal dental follicle (arrow) around the mandibular right third molar (tooth #48). The follicular space measures 2.9 mm (i.e., less than 3 mm). This tooth was subsequently extracted, and no cystic lesion was found in the crown region. (**C**) Panoramic radiograph and (**D**) bone window MDCT image of a patient with a dentigerous cyst (arrow) around the mandibular right third molar (tooth #48). The follicular space measures 4.3 mm (i.e., greater than 3 mm). Histopathological examination after enucleation confirmed the diagnosis of a dentigerous cyst. Permission has been obtained to publish all figures.

**Figure 3:**
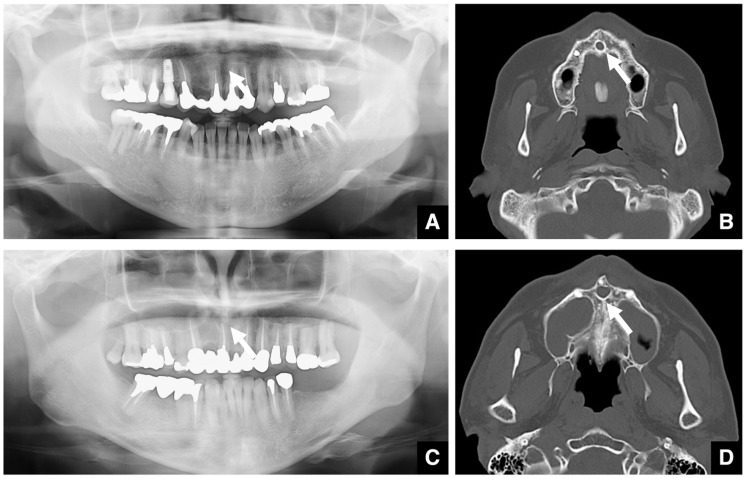
Comparison of nasopalatine duct cyst and normal incisive canal. (**A**) Panoramic radiograph and (**B**) bone window MDCT image (slice thickness, 3.0 mm) of a patient with a normal incisive canal (arrow). The maximum diameter of the canal is 5.9 mm (i.e., within 6 mm). (**C**) Panoramic radiograph and (**D**) bone window MDCT image of a patient with a nasopalatine duct cyst (arrow). The maximum diameter of the canal is 8.4 mm (i.e., greater than 6 mm). Permission has been obtained to publish all figures.

When evaluating impacted teeth, distinguishing between an enlarged dental follicle and a dentigerous cyst can be challenging, especially in the case of maxillary canines and third molars. A dental follicle width of ≤3 mm is considered normal (Figure 2A,B), whereas one of >3 mm suggests a cyst (Figure 2C,D) [7,8]. Similar differentiation applies regarding an enlarged nasopalatine canal versus nasopalatine duct cysts, for which the threshold is the length of the long axis [7,9,10,11]. A nasopalatine canal width of ≤6 mm is considered normal (Figure 3A,B), whereas one of >6 mm suggests a cyst (Figure 3C,D). However, as the nasopalatine canal may widen naturally with age, careful interpretation is required in older patients [9]. In patients over 80 years of age, a nasopalatine canal cyst may be ruled out even if the size exceeds 6 mm. Age correction may be necessary, but no method has yet been developed to indicate the appropriate correction at this time. If only part of the canal is enlarged on sagittal CT, cystic degeneration should be considered. Intraosseous lesions must be managed before surgical intervention.

Maxillary sinusitis has been reported in approximately 60% of patients with jaw deformities [5], evident as mucosal thickening or soft tissue-like structures in the sinus. When sinusitis is present, its treatment should be prioritized before the surgical correction of the jaw deformity. As the mentioned above, in such cases, imaging evaluation using CT, which allows for the assessment of soft tissue lesions, is often effective.

Caries, periodontitis, and associated osteomyelitis must be treated before surgical procedures. In particular, if orthognathic surgery is planned, infections near osteotomy lines must be identified and resolved appropriately.

**Figure 4:**
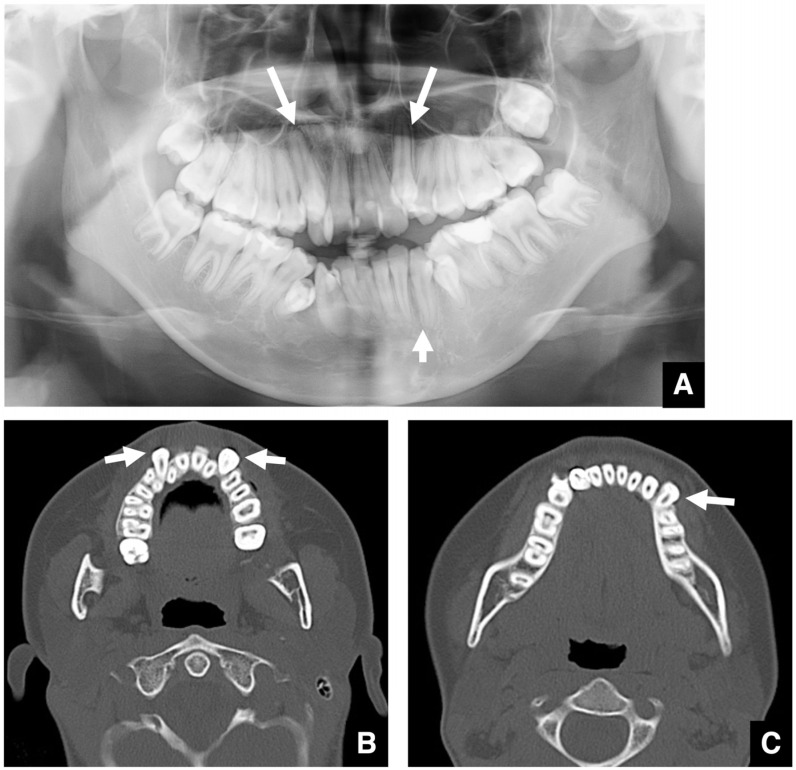
Displacement of maxillary and mandibular canines. (**A**) Panoramic radiograph of a patient with labially displaced and tilted maxillary and mandibular canines (arrows). (**B**) Bone window MDCT image (slice thickness, 3.0 mm) of a patient with bilateral labially displaced and tilted maxillary canines (arrows). (**C**) Bone window MDCT image of a patient with labially displaced and tilted mandibular canine (arrow). Permission has been obtained to publish all figures.

Three-dimensional (3D) evaluation is important in identifying the position and morphology of the teeth, especially the maxillary canines and third molars, in relation to the surrounding anatomical structures. The maxillary canines show variable eruption patterns. When the crown of a maxillary canine is located in the eruption path of a lateral incisor, and the root lies palatally, CT evaluation is essential (Figure 4B,C) [12,13]. Impacted third molars can interfere with orthodontic treatment, sagittal split ramus osteotomy (SSRO), or Le Fort I osteotomy. Therefore, their presence must be assessed preoperatively [13].

### 2.4. Imaging Diagnosis Focused on the Chief Complaint in Jaw Deformities

**Figure 5:**
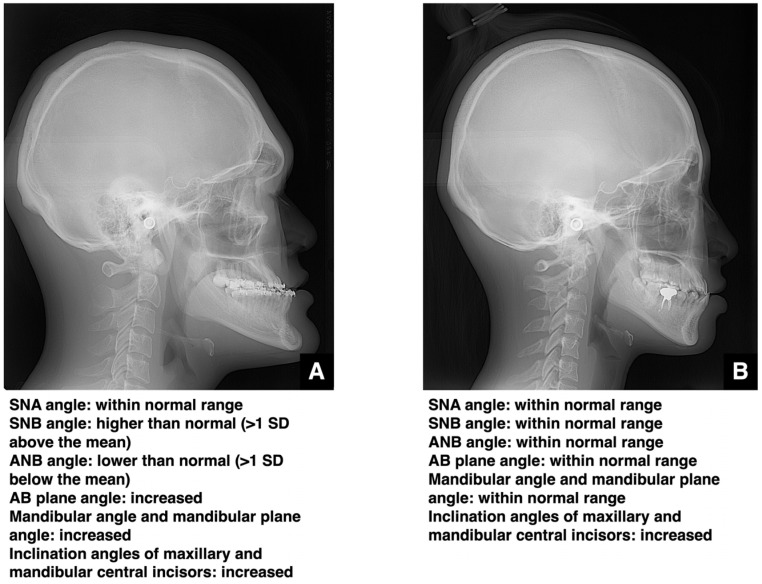
Analysis of skeletal and dentoalveolar mandibular prognathism. (**A**) Lateral cephalometric radiograph and summary of polygonal analysis of a patient with skeletal mandibular prognathism. (**B**) Lateral cephalometric radiograph and summary of polygonal analysis of a patient with dentoalveolar mandibular prognathism. Permission has been obtained to publish all figures.

This section addresses the accurate imaging diagnosis of jaw deformities. Clinically, it is crucial to objectively assess whether the cause of malocclusion is dental or skeletal. Standardized cephalometric radiographs and the various analytical parameters derived from them are commonly used for this purpose. The angle formed between the S-N plane and the nasion of the maxillary alveolar base (SNA angle) and the angle formed between the S-N plane and the nasion of the mandibular alveolar base (SNB angle) are used to evaluate the anteroposterior positions of the maxilla and mandible, respectively. In skeletal mandibular prognathism, the SNA angle remains within the normal range and the SNB angle is more than one standard deviation (SD) above the mean (Figure 5A). In such cases, the findings may raise concern for acromegaly due to mandibular hyperplasia. When the SNA angle is more than one SD below the mean and the SNB angle is within the normal range, skeletal maxillary retrusion is diagnosed. Combined orthognathic surgery and orthodontic treatment is generally considered favorable when the SNB angle is beyond the standard deviation with a standard SNA or when the SNA is beyond the standard deviation with a standard SNB. However, “significantly” is not quantitatively defined, with such decisions currently based on patient preference and the clinical judgment of the orthodontist and oral surgeon. A report that focused only on cases with similar degrees of skeletal disharmony and compared in detail 10 cases of orthodontic treatment alone with 10 cases of surgical orthodontic treatment within the range of ANB −1 to −5° and Wits −6 to −13 mm reported that combining ANB, Wits, the anterior tooth axis, and soft tissue evaluation demonstrated the high utility of surgical orthodontic treatment [14]. Furthermore, a study comparing 13 cases of adult skeletal mandibular protrusion treated with orthodontics alone and 12 cases treated with surgical orthodontics identified a Holdaway angle ≥ 12° as the threshold where orthodontics alone was more likely to be selected, while angles < 12° favored surgical orthodontics [15]. Based on these findings, it is considered that cases meeting three or more of the following criteria for skeletal mandibular prognathism could serve as a guideline: ANB angle ≤ 1°, Wits appraisal ≤ −4 mm, overjet ≤ 0 mm, and molar relationship Class III or higher. Furthermore, the range of ANB angles between −1° and −5° and Wits appraisal values between −6 mm and −13 mm are considered borderline areas where both orthodontic treatment alone and surgical orthodontic treatment are clinically viable options [14]. However, even within these ranges, if there is a strong compensatory inclination of the mandibular incisors and limited potential for further alveolar compensation, and if there is a high demand for improvement in the soft tissue facial profile (e.g., prominent chin, receding lower lip) (reference indicator: Holdaway angle < 12°), surgical orthodontic treatment (orthognathic surgery) should be considered as the first choice [14].

Although it may not be possible to adequately correct severe skeletal deformities by orthodontic treatment alone, surgical intervention should not be imposed on patients who do not desire it.

**Figure 6:**
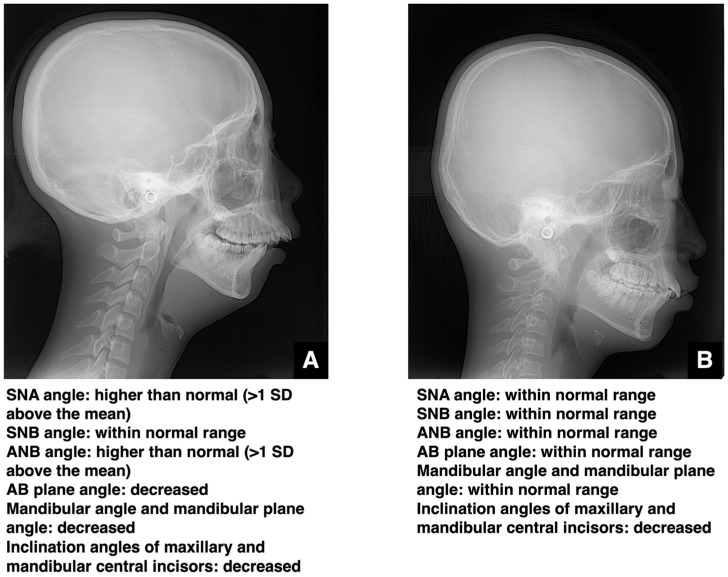
Analysis of skeletal and dentoalveolar maxillary protrusion. (**A**) Lateral cephalometric radiograph and summary of polygonal analysis of a patient with skeletal maxillary protrusion. (**B**) Lateral cephalometric radiograph and summary of polygonal analysis of a patient with dentoalveolar maxillary protrusion. Permission has been obtained to publish all figures.

In skeletal maxillary prognathism, the SNA is more than one SD above the mean, and the SNB remains normal (Figure 6A). When the SNA angle is within the normal range and the SNB angle is more than one SD below the mean, skeletal mandibular retrusion is diagnosed. As with mandibular prognathism and maxillary retrusion, the choice between orthodontic treatment alone and combined surgical–orthodontic intervention depends on the patient’s wishes and the experience of the clinical team. A comparative study of 40 cases successfully treated with orthognathic surgery, 40 cases successfully treated with orthodontics alone, and 21 cases deemed unsuccessful with orthodontic treatment alone reported that, in adolescent patients with Class II malocclusion past the growth phase, particularly those with a mandibular protrusion of 10 mm or more, a vertical distance from the mandibular symphysis to the nasal root of 18 mm or more, a mandibular body length less than 70 mm, or a facial height greater than 125 mm, orthodontic treatment alone is likely to fail and surgical intervention is highly recommended for the successful correction of the malocclusion [16]. The decision criteria regarding whether to combine surgical procedures in the treatment of jaw deformities involve significant patient input and cannot be determined by numerical values alone.

**Figure 7:**
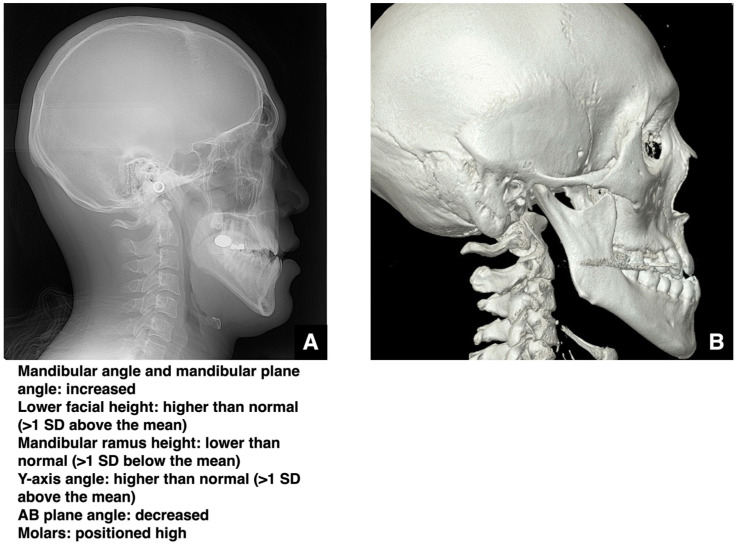
Analysis of skeletal mandibular prognathism with open bite. (**A**) Lateral cephalometric radiograph and summary of polygonal analysis of a patient with skeletal mandibular prognathism and open bite. (**B**) Reconstructed MDCT image (slice thickness, 0.5 mm) of a patient with skeletal mandibular prognathism and open bite. Permission has been obtained to publish all figures.

In cases of skeletal open bite, the mandibular plane angle is elevated (more than one SD), and the lower facial height tends to be increased, whereas the height of the mandibular ramus is often reduced (Figure 7A,B). In some cases, the maxilla exhibits superior rotation, indicated by a larger Ar-PNS-ANS angle. Treatment choices follow the same principles as for other skeletal deformities. Open bite accompanied by skeletal mandibular prognathism is typically harder to manage with orthodontics alone compared to isolated prognathism. When selecting a surgical method, the degree of jaw movement and likelihood of relapse must be carefully considered. For example, even if open bite correction by SSRO is initially successful, relapse due to mandibular rotation can occur within a few years [17,18,19].

Yoshioka et al. reported that, in skeletal mandibular prognathism with an open bite ≥3 mm, SSRO alone resulted in a relapse of about 1.5 mm after one year [20]. For open bites of <3 mm, relapse was approximately 0.5 mm. However, when combining Le Fort I osteotomy with SSRO, even in open bites ≥ 3 mm, relapse was only about 0.6 mm [20]. Thus, for skeletal mandibular prognathism with open bite, the surgery type should be chosen based on whether the open bite is ≥3 mm or <3 mm. However, the authors of the mentioned paper state that the sample size was small and did not allow the analysis of the oral surgeon’s skill, patient sensitivity, or the degree of surgical correction [20]. In patients with skeletal mandibular protrusion and open bite, the vertical movement of the mandibular angle should be set large after SSRO, and the pterygoid muscle sling should be fixed tightly [21,22]. Particularly when the vertical movement of the mandibular angle exceeds 3 mm, the pterygoid muscle sling must be secured significantly more tightly after SSRO. Apart from the report by Yoshioka et al., no paper has presented selection criteria for the combined use of SSRO and Le Fort I versus SSRO alone, and our analysis at this institution has not revealed any major issues [20]. Future studies should aim to validate the diagnostic criteria.

Because cephalometric radiographs are two-dimensional (2D), they have limited utility in evaluating the 3D morphology of the maxillofacial skeleton, particularly differences in depth between the left and right sides. Hajeer et al. described the potential applications of 3D imaging using CT/CBCT in orthodontic diagnosis and treatment, highlighting the advantages of 3D modalities over 2D imaging for orthodontic and orthognathic assessment [23]. Regarding the discussion of the limitations of 2D imaging and the transition to 3D digital models to evaluate the maxillofacial skeleton, previous studies have demonstrated that three-dimensional measurements, such as the distances between landmarks and angles formed by landmarks, possess sufficient accuracy for clinical applications [24,25,26,27]. On the other hand, a meta-analysis also exists indicating that landmarks on the mid-sagittal line and dental landmarks show the highest reliability, while landmarks on the condyle, frontal eminence, and orbital eminence have lower reliability [28]. The same paper demonstrated that multiplanar images related to visualization via 3D reconstruction require marking the S point [28]. Furthermore, it concludes that additional research is needed for the evaluation of soft tissue landmarks [28].

**Figure 8:**
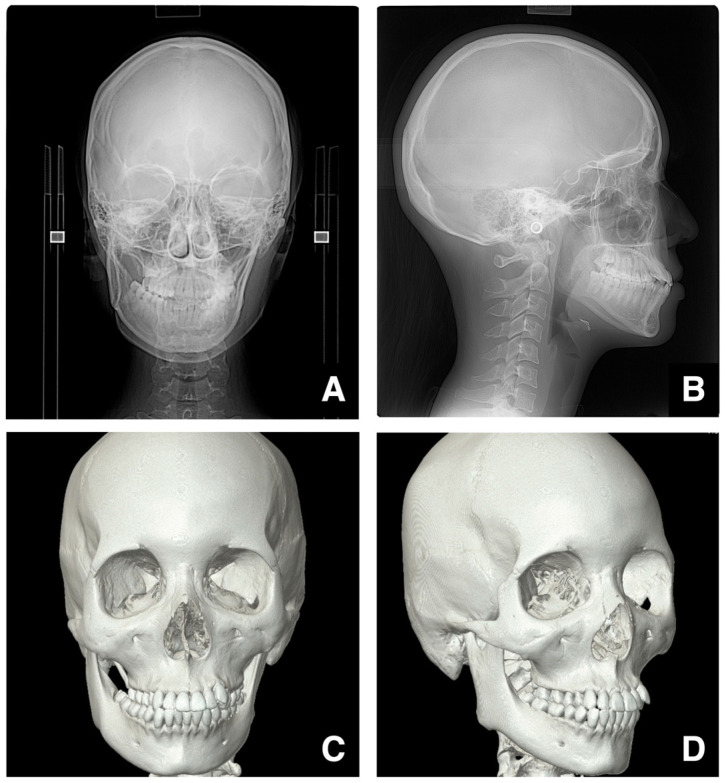
Three-dimensional reconstructed images using CT data and cephalometric radiographs in skeletal mandibular prognathism with facial asymmetry. (**A**) Frontal cephalometric radiograph of a patient with skeletal mandibular prognathism and facial asymmetry. (**B**) Lateral cephalometric radiograph of the same patient. It is difficult to evaluate the right and left sides of the craniofacial skeleton separately. (**C**) Frontal view of a 3D reconstructed image created from MDCT image data (slice thickness, 0.5 mm) of the same patient. (**D**) Right oblique view of the same 3D reconstructed image. Permission has been obtained to publish all figures.

In particular, 3D images generated from CT data are highly useful in accurately diagnosing jaw deformities [29,30,31,32] and particularly valuable in cases of facial asymmetry or open bite (Figure 8A–D) and for less experienced clinicians.

The 3D reconstructed images allow the separate evaluation of the right and left sides of the craniofacial skeleton and improve the understanding of spatial relationships. Hajeer et al. reported the use of 3D imaging techniques, which capture the full facial geometry, to register and analyze facial structures in surgical planning [33,34], including the measurement of the amount of movement required on each side during orthodontic treatment or surgery.

In cases of facial asymmetry, the mid-sagittal alignment of the upper and lower dental arches should be checked first, followed by the assessment of differences in vertical height between the left and right palatal or occlusal planes. CT-based 3D imaging allows the simulation of the required jaw movements, including measurements for each side (Figure 8C,D).

Furthermore, while 3D imaging is useful for spatial diagnosis, its application in surgical planning should extend to the objective assessment of soft tissue and volumetric changes. To provide a comprehensive diagnostic workup, clinicians should utilize validated 3D methods to quantify these changes postoperatively [35].

**Figure 9:**
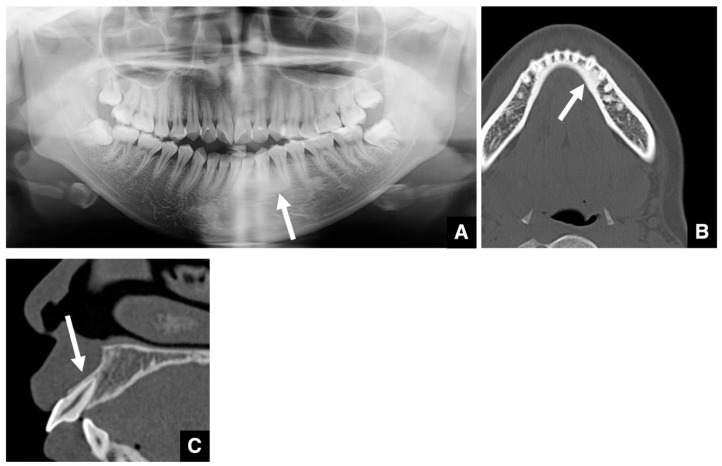
Evaluation of the relationship between tooth roots and surrounding bone structures in planning for orthodontic movement. (**A**) Panoramic radiograph of a patient with enostosis (arrow). (**B**) Axial bone window MDCT image (slice thickness, 3.0 mm) of the region with enostosis (arrow). The relationship between the tooth root and the enostosis should be evaluated, and root movement should be planned to avoid the lesion. (**C**) Sagittal bone window MDCT image (slice thickness, 2.0 mm) shows contact between the cortical bone and the tooth root (arrow). Attention should be paid to the relationship between the cortical bone and the root to avoid applying pressure on the cortical bone during tooth movement. Permission has been obtained to publish all figures.

During tooth movement, contact with cortical bone or the presence of dense bone islands can cause root resorption or exposure. Thus, imaging evaluation is necessary to assess the spatial relationship between the roots and cortical bone, as well as the presence and extent of any bone islands. When the roots are adjacent to the cortical bone, or if bone islands are present, root movement should avoid these regions [36]. Areas of increased radiopacity on panoramic or periapical images should be evaluated further with CT to detect bone islands (Figure 9A–C).

Longitudinal changes in jaw growth are evaluated on cephalometric radiographs to confirm whether mandibular growth has ceased. Growth cessation is further assessed by examining the bone age on hand–wrist radiographs. In some cases, a bone scan may be performed to assess growth cessation based on the degree of uptake at the metaphysis. If growth cessation cannot be determined, surgery may need to be delayed until growth cessation can be confirmed. Even then, relapse may occur after surgery. Factors such as the influence of muscles and soft tissues, poor fixation during surgery, effects on the temporomandibular joint, including mandibular condyle resorption, and ongoing growth may be considered.

### 2.5. Confounding Factors/Limitations

This review paper presents representative cases in a narrative format, drawing on general textbook content and the practical experience of the authors’ institution; it is not a systematic review. Therefore, there is a possibility of bias in the content described and the literature cited. However, by presenting the clinical context at our institution as one example, supported by the literature, we believe that this approach can serve as a reference point for perspectives from other institutions and, conversely, offer an opportunity to learn about emerging practices.

### 2.6. Imaging Evaluation of the TMJ in Jaw Deformities

Detailed evaluation of the TMJ is performed using panoramic radiographs, four-segment panoramic TMJ images, CT including CBCT, and MRI [37,38,39,40]. Sagittal and 3D reconstructions of CT data are particularly useful for the evaluation of hard tissue structures [37,38].

**Figure 10:**
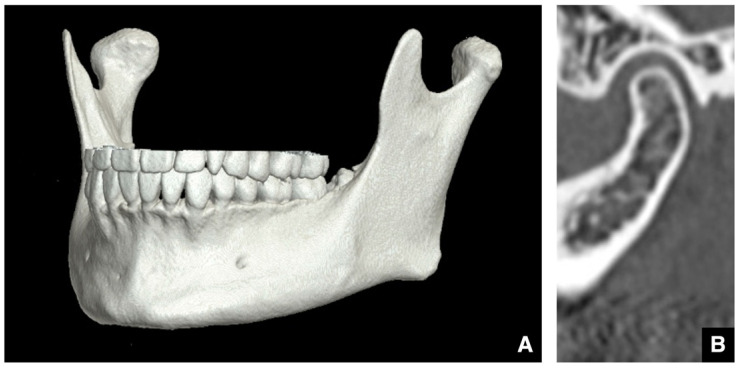
CT images of normal temporomandibular joints. (**A**) A 3D reconstructed image created from MDCT image data (slice thickness, 0.5 mm) shows normal mandibular condyles. (**B**) Corrected sagittal MDCT image shows a normal temporomandibular joint. Permission has been obtained to publish all figures. Created from CT data.

**Figure 11:**
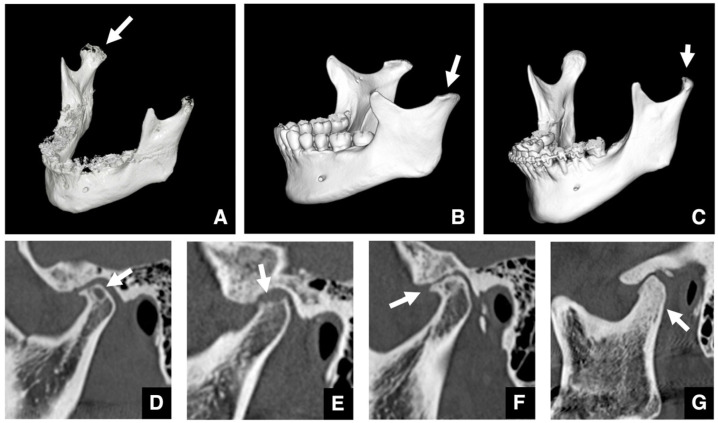
MDCT images of osteoarthrosis of the temporomandibular joint. (**A**) Three-dimensional reconstructed images created from MDCT data (slice thickness, 0.5 mm) show a depression in the mandibular condyle (arrow), (**B**) 3D flattening of the mandibular condyle (arrow), and (**C**) narrowing of the mandibular condyle (arrow). MDCT corrected (**D**) sagittal images (slice thickness, 2.0 mm) show a subchondral cyst (Ely’s cyst) in the mandibular condyle (arrow), (**E**) erosive changes in the cortical bone of the mandibular condyle (arrow), (**F**) osteophyte formation on the cortical surface of the mandibular condyle (arrow), and (**G**) a generalized sclerotic change in the mandibular condyle (arrow). Permission has been obtained to publish all figures.

In the normal TMJ, the condyle, glenoid fossa, and articular eminence exhibit well-matched curvature. Internally, these structures are surrounded by a continuous cortical bone layer and show a uniform trabecular bone pattern (Figure 10A,B). In contrast, abnormalities in the TMJ may present as condylar narrowing or flattening and the flattening of the glenoid fossa or articular eminence (Figure 11A–C). Internally, cortical bone thinning or loss, erosive changes, subchondral cysts, osteophyte formation, and generalized sclerotic changes in trabecular bone may be observed (Figure 11D–G). In patients with rapidly progressing symptoms, progressive condylar resorption (PCR) should be considered.

**Figure 12:**
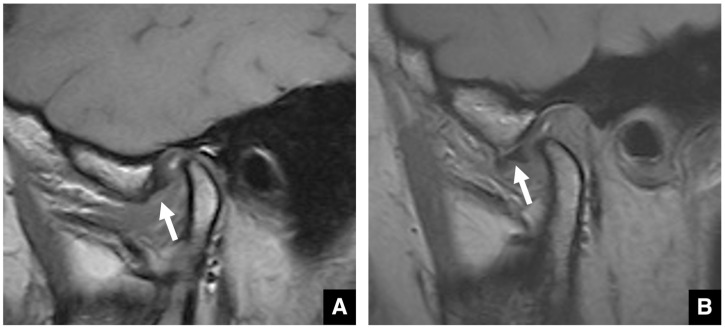
MR images of anterior disc displacement without reduction in the temporomandibular joint. (**A**) T1-weighted images (TR 1050 ms, TE 18 ms, slice thickness 3.0 mm) show anterior displacement of the articular disc (arrow) in the closed-mouth position and (**B**) persistent anterior displacement of the disc, without reduction (arrow), in the open-mouth position.

**Figure 13:**
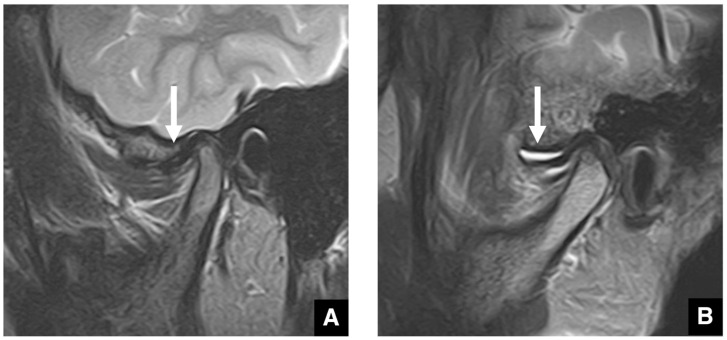
Comparison of a normal temporomandibular joint and a joint with TMJ effusion. In closed-mouth T2-weighted MR images (TR 4000 ms; TE 108 ms; slice thickness 3.0 mm), (**A**) no areas of high signal indicative of fluid are observed in the joint space (arrow), and (**B**) a high-signal area indicating TMJ effusion is observed in the superior joint space (arrow). Permission has been obtained to publish all figures.

Soft tissue evaluation of the TMJ, including the articular disc, is best performed by MRI. Conditions such as anterior disc displacement with or without reduction are diagnosed by evaluating the positional relationship between the disc and condyle on T1-weighted images (Figure 12A,B). T2-weighted images are additionally used to confirm TMJ effusion (Figure 13B).

**Figure 14:**
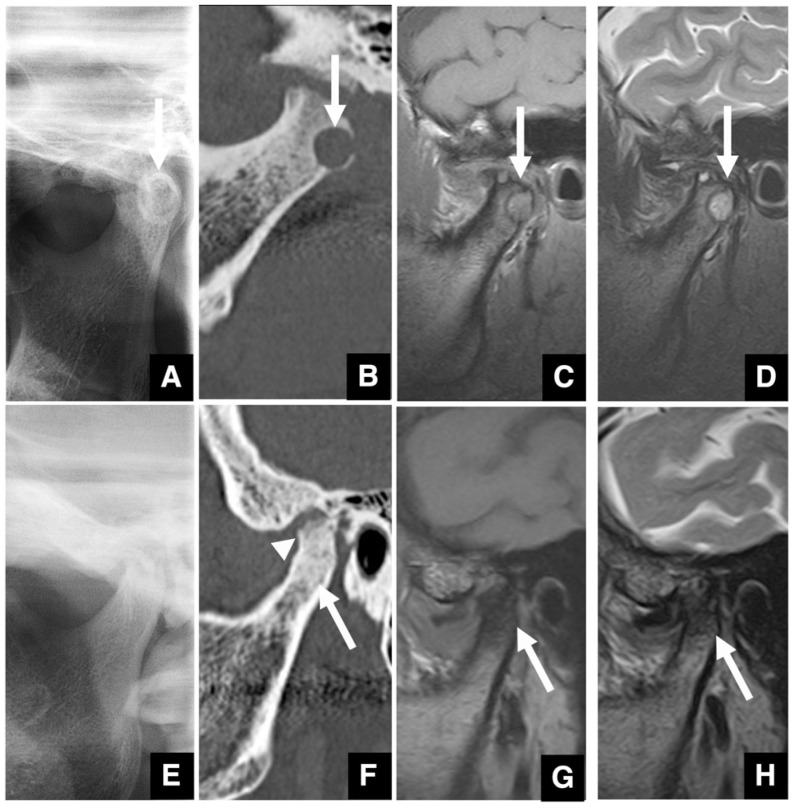
Imaging findings of osteoarthrosis of the temporomandibular joint. (**A**) Panoramic radiograph of a patient with a subchondral cyst and sclerotic changes (arrow) in the mandibular condyle. (**B**) MDCT corrected sagittal image (slice thickness, 2.0 mm) of the same patient as in (**A**) shows a subchondral cyst (arrow) and sclerotic changes in the mandibular condyle. MRI T1-weighted image (TR 1050 ms, TE 18 ms, slice thickness 3.0 mm) of the same patient as in (**A**). (**C**) The signal intensity of the subchondral cyst (arrow) in the mandibular condyle is higher than that of the muscle. MRI T2-weighted image (TR4000 ms; TE 108 ms; slice thickness 3.0 mm) of the same patient as in (**A**). (**D**) The subchondral cyst (arrow) in the mandibular condyle shows high signal intensity. (**E**) Panoramic radiograph and (**F**) MDCT corrected sagittal image of a patient with sclerotic changes (arrow) in the mandibular condyle, glenoid fossa, and articular eminence and erosive changes (arrowhead) in the mandibular condyle. (**G**) MRI T1-weighted image and (**H**) MRI T2-weighted image of the same patient as in (**E**). The mandibular condyle shows no signal (arrow) throughout. Permission has been obtained to publish all figures.

Lesions that appear as subchondral cysts on CT may show a high signal intensity on both T1- and T2-weighted MRI compared to the surrounding muscle tissue (Figure 14A–D). Differential diagnoses may include jaw cysts such as simple bone cysts, aneurysmal bone cysts, and related entities. Generalized sclerosis is characterized by the disappearance of trabecular patterns and increased radiodensity on CT, while showing low signals on both T1- and T2-weighted MRI (Figure 14E–H). In such cases, the cortical bone margins may become indistinct and irregular, sometimes accompanied by periosteal reactions. There is the absence of a signal in the mandibular condyle on MRI due to marked sclerotic changes throughout the entire condyle, based on the CT appearance.

**Figure 15:**
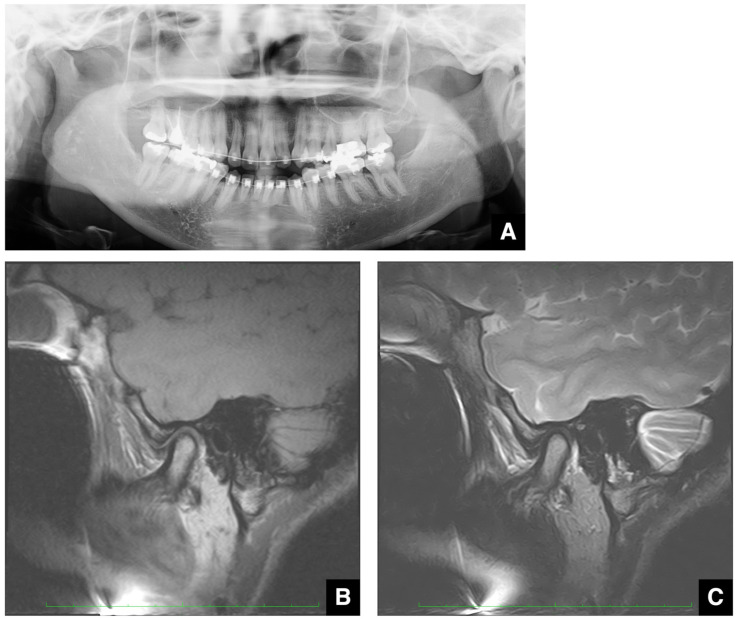
Images of a patient undergoing orthodontic treatment with archwires in place. (**A**) Panoramic radiograph shows the archwires. (**B**) T1-weighted MR image (TR 1050 ms; TE 18 ms; slice thickness 3.0 mm) of the same patient as in (**A**). (**C**) T2-weighted MR image (TR 4000 ms; TE 108 ms; slice thickness 3.0 mm) of the same patient as in (**A**). In both the T1- and T2-weighted images, artifacts obliterate the jawbone region but do not affect the visualization of the temporomandibular joint. Permission has been obtained to publish all figures.

TMJ disorders can develop postoperatively after surgery for the correction of jaw deformities [41,42]. To minimize such complications, preoperative evaluation of the TMJ using MRI is recommended. MRI of the TMJ can be performed in patients undergoing orthodontic treatment as long as the archwires are removed, because the TMJ region is less prone to image distortion from metallic artifacts (Figure 15B,C). Placing the surface coil on the skin near the temporomandibular joint additionally helps to reduce artifacts. Thus, MRI of the TMJ is feasible in many patients during orthodontic treatment.

### 2.7. Imaging Evaluation to Prevent Complications During Jaw Deformity Surgery

This section discusses the imaging evaluations necessary to prevent complications associated with surgery. Representative surgical techniques include SSRO, genioplasty, and Le Fort I osteotomy.

When performing SSRO or IVRO, it is critical to understand the relevant soft tissue and bony structures. Important soft tissue structures to consider include the maxillary artery, facial artery and vein, retromandibular vein, and facial nerve [43,44]. Measuring the approximate distances of these vessels and nerves from the lateral, posterior, and inferior borders of the mandible on imaging may help to avoid intraoperative injury.

Careful evaluation of intraosseous structures is also necessary for SSRO (Table 2) [45]. The course of the mandibular canal and the surrounding bone quality must be assessed, especially in the region where the proximal and distal bone segments are created. The canal often deviates lingually near the second molar, but individual variation exists in its buccolingual transition [46,47,48]. Careful examination of cross-sectional CT images is essential.

**Figure 16:**
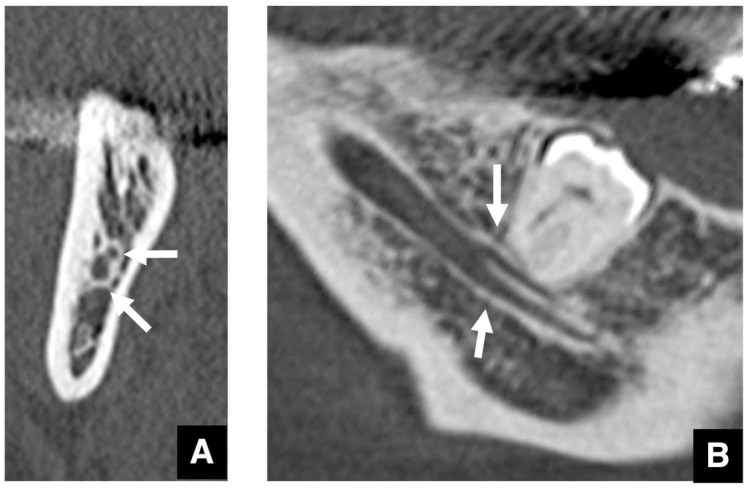
Bifurcation of the mandibular canal distal to the mandibular third molar. (**A**) Two mandibular canals (arrows) are seen on an MDCT cross-sectional image (slice thickness, 2.0 mm) at the level distal to the mandibular third molar. (**B**) Panoramic section image (slice thickness, 2.0 mm) shows bifurcation of the mandibular canal into two branches (arrows) distal to the mandibular third molar. Permission has been obtained to publish all figures.

Bifid or trifid inferior alveolar nerves occur in approximately 20–50% of patients (Figure 16A,B) [49,50,51]. If such branching is visible on CT, the course of each branch must be clearly identified. Preoperative evaluation of the mandibular canal and bone quality also helps to predict the risk of postoperative inferior alveolar nerve paresthesia [47,48,52]. Nerve injury is more likely in the case of a short distance from the buccal wall of the mandibular canal to the buccal cortical margin near the second molar (the typical osteotomy site) and if the bone is dense. In addition, because females are more prone to sensory disturbances after SSRO [47,48,52], more detailed explanations are required for female patients during surgery and postoperative follow-up.

**Figure 17:**
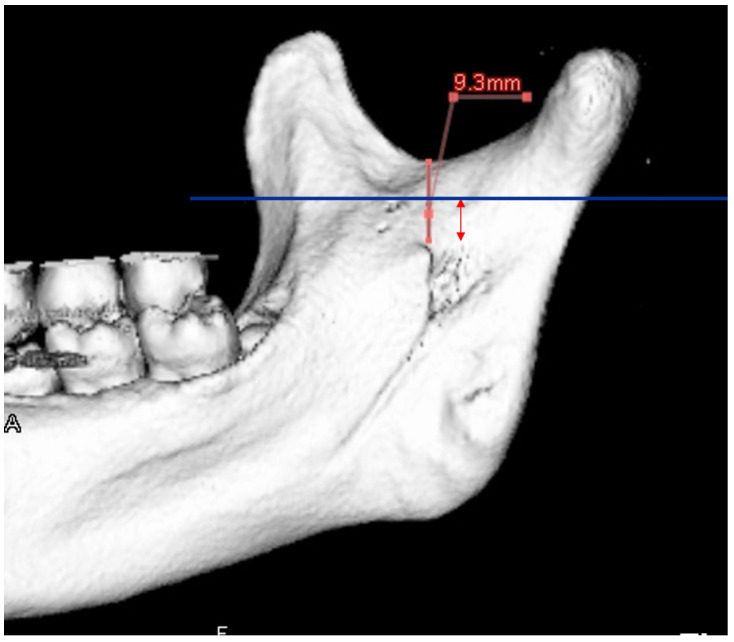
A 3D reconstructed image created from MDCT data (slice thickness, 0.5 mm) for measurement purposes prior to SSRO. The distance from the mandibular notch to the lingula should be at least 14 mm (arrow). Permission has been obtained to publish the figure.

Before SSRO, it is important to measure the distance from the mandibular notch to the lingula using 3D CT. A minimum distance of 14 mm is considered necessary (Figure 17) [53]. The position of the lingula varies, and its relationship with the antelingula is debated [54,55]. Therefore, 3D images created using CT data are extremely useful [23,33]. Based on the literature and our clinical experience, protocols specific to the authors’ institution are presented.

**Figure 18:**
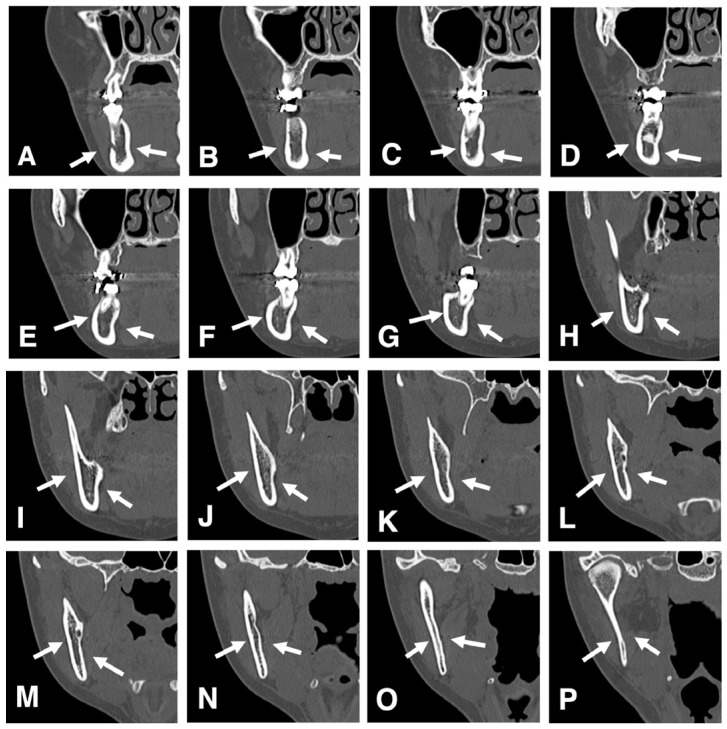
Evaluation of cortical thickness at the medial and lateral osteotomy regions. Consecutive thin-slice MDCT images (slice thickness, 2.0 mm) in the coronal place show the buccolingual cortical bone thickness (**A**) from the mental foramen (**P**) to the condylar head of the mandible. The thickness of each of the buccal and lingual cortical plates of the mandible (arrows) varies depending on the location. Permission has been obtained to publish all figures (**A**–**P**). (**A**) Mandibular buccal and lingual cortical bone (arrows) are seen on an MDCT cross-sectional image (slice thickness, 2.0 mm) at the level of mental foramen. (**B**) Mandibular buccal and lingual cortical bone (arrows) are seen on an MDCT cross-sectional image (slice thickness, 2.0 mm) at the level distal to (**A**). (**C**) Mandibular buccal and lingual cortical bone (arrows) are seen on an MDCT cross-sectional image (slice thickness, 2.0 mm) at the level of mandibular first molar. (**D**) Mandibular buccal and lingual cortical bone (arrows) are seen on an MDCT cross-sectional image (slice thickness, 2.0 mm) at the level distal to (**C**). (**E**) Mandibular buccal and lingual cortical bone (arrows) are seen on an MDCT cross-sectional image (slice thickness, 2.0 mm) at the level distal to (**D**). (**F**) Mandibular buccal and lingual cortical bone (arrows) are seen on an MDCT cross-sectional image (slice thickness, 2.0 mm) at the level of mandibular second molar. (**G**) Mandibular buccal and lingual cortical bone (arrows) are seen on an MDCT cross-sectional image (slice thickness, 2.0 mm) at the level distal to (**F**). (**H**) Mandibular buccal and lingual cortical bone (arrows) are seen on an MDCT cross-sectional image (slice thickness, 2.0 mm) at the level of the retromolar region. (**I**) Mandibular buccal and lingual cortical bone (arrows) are seen on an MDCT cross-sectional image (slice thickness, 2.0 mm) at the level distal to (**H**). (**J**) Mandibular buccal and lingual cortical bone (arrows) are seen on an MDCT cross-sectional image (slice thickness, 2.0 mm) at the level distal to (**I**). (**K**) Mandibular buccal and lingual cortical bone (arrows) are seen on an MDCT cross-sectional image (slice thickness, 2.0 mm) at the level of mandibular ramus. (**L**) Mandibular buccal and lingual cortical bone (arrows) are seen on an MDCT cross-sectional image (slice thickness, 2.0 mm) at the level of mandibular foramen. (**M**) Mandibular buccal and lingual cortical bone (arrows) are seen on an MDCT cross-sectional image (slice thickness, 2.0 mm) at the level distal to (**L**). (**N**) Mandibular buccal and lingual cortical bone (arrows) are seen on an MDCT cross-sectional image (slice thickness, 2.0 mm) at the level distal to (**M**). (**O**) Mandibular buccal and lingual cortical bone (arrows) are seen on an MDCT cross-sectional image (slice thickness, 2.0 mm) at the level distal to (**N**). (**P**) Mandibular buccal and lingual cortical bone (arrows) are seen on an MDCT cross-sectional image (slice thickness, 2.0 mm) at the level of the mandibular condyle.

The cortical thickness at the medial and lateral osteotomy regions should also be evaluated (Figure 18A–P). The osteotomy line is placed in an area where the mandibular canal shifts from the buccal to the lingual side and the buccal cortical bone is thin. The buccal cortical bone must also be evaluated for thickness, especially at the inferior border, as the osteotomy must extend slightly to the lingual cortex to ensure complete division of the bone [48]. By understanding the precise variations in cortical bone plate thickness in advance, as identified on CT and incorporated into the surgical plan, smoother implementation becomes possible.

**Figure 19:**
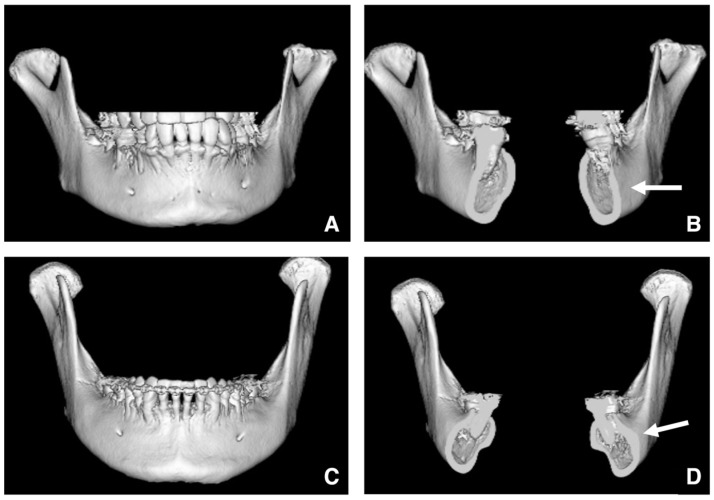
Evaluation of lateral bulging of the mandibular angle. Three-dimensional reconstructed images created from MDCT data (slice thickness, 0.5 mm) of (**A**) the mandible and (**B**) mandibular ramus regions show normal anatomy. The mandibular angle is concave, providing a clear view of the inferior border (arrow). Three-dimensional reconstructed images created from MDCT data of (**C**) the mandible and (**D**) mandibular ramus regions show excessive lateral bulging. The visibility of the inferior border is reduced (arrow) where buccal bulging is pronounced. Permission has been obtained to publish all figures.

The lateral bulge of the mandible is best visualized on 3D CT images. Typically, the inferior border is well visualized in patients with minimal lateral bulging of the mandible (Figure 19A,B). However, excessive lateral bulging can reduce the visibility during buccal osteotomy (Figure 19C,D).

**Figure 20:**
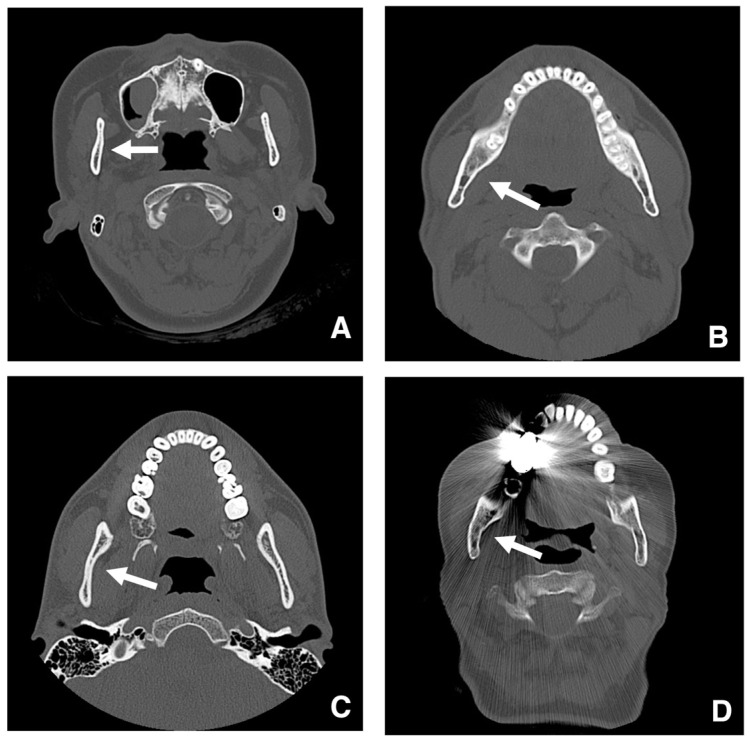
Evaluation of the degree of medial curvature of the mandibular ramus. In axial bone window MDCT images (slice thickness, 3.0 mm) at the level of (**A**) the mandibular notch and (**B**) the mandibular body, the mandibular ramus appears straight (arrows). In axial bone window MDCT images at the level of (**C**) the mandibular notch and (**D**) the mandibular body, the mandibular ramus shows marked curvature (arrows). Permission has been obtained to publish all figures.

The degree of curvature on the medial side of the ramus should also be evaluated on CT. A straight ramus poses no problem (Figure 20A,B), but strong curvature complicates visualization and the insertion of cutting tools during lingual osteotomy (Figure 20C,D) [48]. It has been suggested that a highly curved ramus correlates with longer operative times and greater blood loss [56].

**Figure 21:**
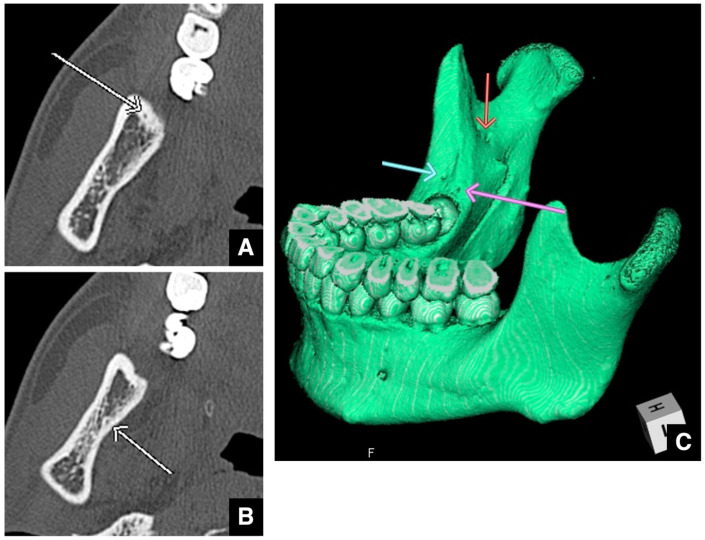
CT images show the course of small blood vessels from the mandibular molar region to the area around the mandibular notch. Axial bone window MDCT images (slice thickness, 3.0 mm) show small blood vessels (**A**) in the posterior mandibular molar region and (**B**) near the mandibular notch. The vessels appear as tubular defects (arrows). A 3D reconstructed image created from MDCT data of the same patient shows small blood vessels in the posterior mandibular molar region and around the mandibular notch. (**C**) The vessels appear as depressions (arrows). Permission has been obtained to publish all figures.

To minimize bleeding, small nutrient vessels in the cortical bone surrounding the osteotomy site should be evaluated. These vessels often appear as depressions or canal-like defects near the posterior molar and ramus regions (Figure 21A,B). In particular, 3D imaging can assist in the identification of such structures (Figure 21C). By identifying the precise course of nutrient vessels from CT-based anatomical assessment and integrating this information into the surgical plan, smoother preemptive hemostasis can be achieved.

**Figure 22:**
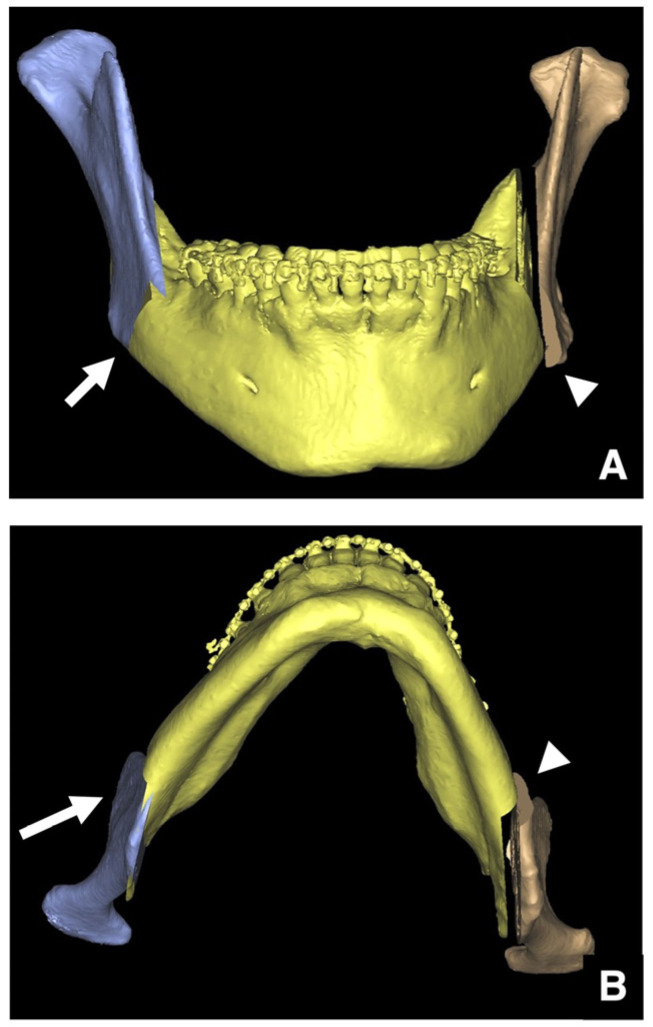
Relationship between proximal and distal bone segments in a simulation image after SSRO. (**A**) Frontal view of a 3D reconstructed simulation image created using MDCT data (slice thickness, 0.5 mm). Interference between the distal and proximal bone segments is observed on the right side (arrow), whereas a gap is present on the left side, with no interference. (**B**) The same simulation image viewed from the inferior aspect. Interference between the distal and proximal bone segments is observed on the right side (arrow), whereas a gap is present on the left side, with no interference. Permission has been obtained to publish all figures.

Finally, simulation software (ProPlan CMF, Materialise, Leuven, Belgium) using 3D CT data can be used to predict interference between the proximal and distal segments (Figure 22A,B). If interference is expected, adjustments to the osteotomy site or surgical method may be necessary [29].

**Figure 23:**
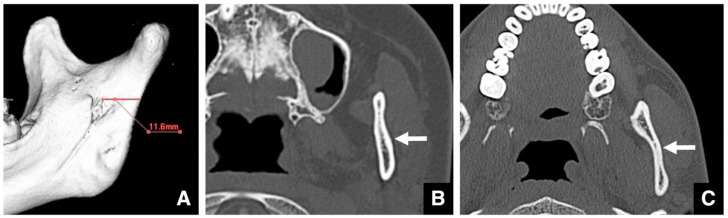
MDCT images illustrating mandibular anatomical considerations in planning for IVRO. (**A**) The shortest distance from the posterior border of the mandible to the mandibular foramen is measured on a 3D image created from MDCT data (slice thickness, 0.5 mm). A distance of ≥5–7 mm is the standard guideline for the vertical osteotomy line in IVRO. (**B**) The mandibular ramus appears straight (arrow) on an axial bone window MDCT image (slice thickness, 3.0 mm) at the level of the mandibular notch. Mucosal cysts are seen incidentally in the left maxillary sinus. (**C**) The mandibular ramus shows marked curvature (arrow) on an axial bone window MDCT image at the level of the mandibular notch. Permission has been obtained to publish all figures.

For IVRO, CT evaluation focuses on the distance from the posterior mandibular border to the mandibular foramen [48]. A vertical osteotomy line at least 5–7 mm anterior to the posterior border is recommended. The buccolingual width and curvature of the ramus should also be evaluated, as a wider and more curved ramus requires a longer saw blade. Deeper insertion increases a risk of injury to the maxillary artery (Figure 23A,C).

### 2.8. Imaging Evaluation for Genioplasty

Table 3 lists the soft tissue and bony structures that must be carefully evaluated prior to genioplasty [57,58]. Among these, the key soft tissue structures to consider are the submental artery, sublingual artery, and mental nerve.

**Figure 24:**
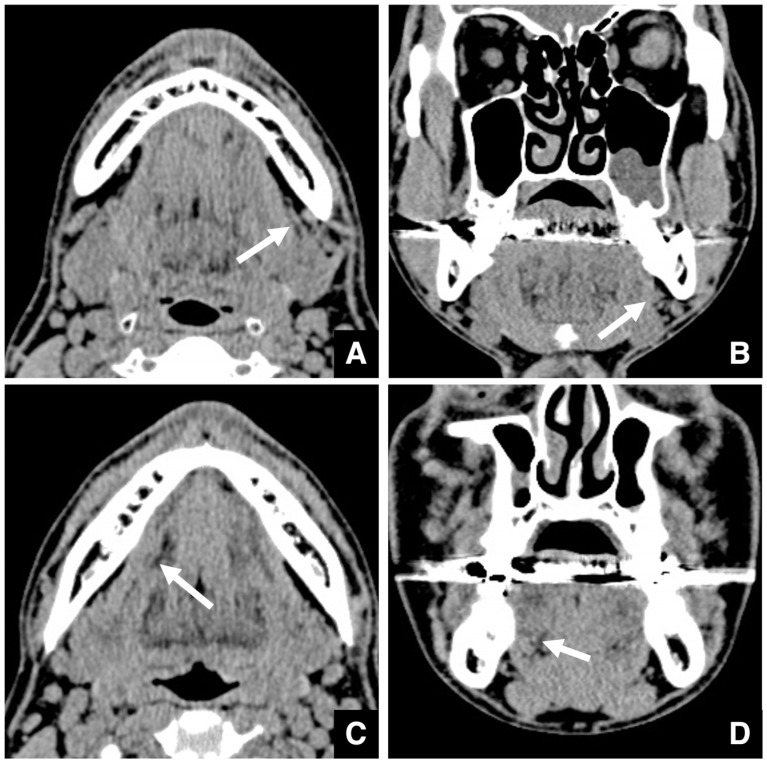
MDCT images (slice thickness, 3.0 mm) showing the submental and sublingual arteries. (**A**) Axial MDCT image at the level of the inferior border of the mandible shows the submental artery (arrow). (**B**) Coronal MDCT image at the level of the hyoid bone shows the submental artery (arrow) running medial to the submandibular line. Mucosal cysts are seen incidentally in the left maxillary sinus. (**C**) Axial MDCT image at the mandibular level shows the sublingual artery (arrow). (**D**) Coronal MDCT image at the level of the molars shows the sublingual artery (arrow) running through fat tissue in the submental region. Permission has been obtained to publish all figures.

The submental artery is located medial to the submandibular line (Figure 24A,B). The sublingual artery can be identified by observing fat tissue in the submental region (Figure 24C,D). Measuring the distances from these vessels to the lateral, posterior, and inferior borders of the mandible can help to avoid injury during surgery.

The mental nerve exits from the mental foramen and travels toward the lower lip, labial gingiva, and buccal mucosa [59]. However, its visualization in standard imaging is limited.

**Figure 25:**
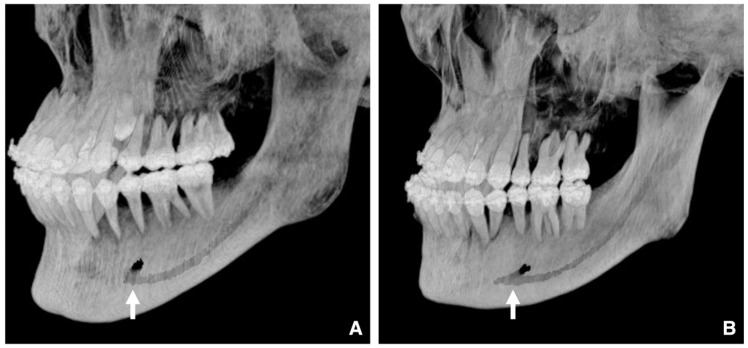
Three-dimensional MDCT images demonstrating the course of the mandibular canal. (**A**) The mandibular canal forms an anterior loop of approximately 3 mm before opening at the mental foramen (arrow). (**B**) In rare cases, the anterior loop may be longer; in this case, it measures approximately 10 mm (arrow). Permission has been obtained to publish all figures.

In terms of intraosseous structures (Table 4) [60,61], the osteotomy line must be placed anterior to the mental foramen. The position of the mental foramen and the course of the incisive branch must be confirmed. In many cases, the mandibular canal forms a loop of 2–3 mm before opening at the mental foramen, and, in rare cases, it may extend up to 10 mm (Figure 25) [62]. The root positions of the mandibular anterior teeth, the mandibular morphology, and the cortical bone thickness at each site should be evaluated to safely form and secure the bone segments [63].

### 2.9. Imaging Evaluation for Le Fort I Osteotomy

**Figure 26:**
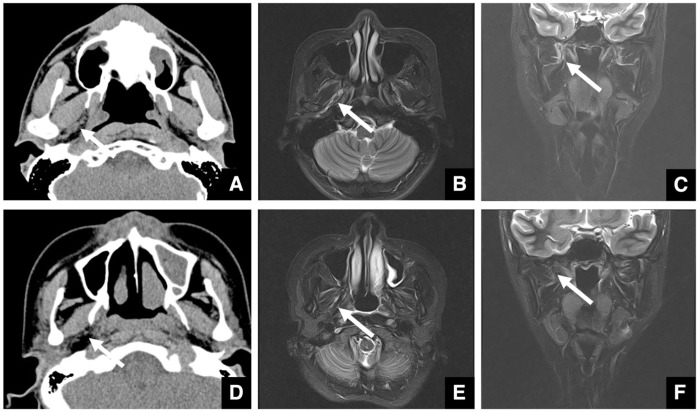
MDCT (slice thickness, 3.0 mm) and MR images of the pterygoid venous plexus. (**A**) Axial soft tissue MDCT image of a well-developed pterygoid venous plexus. Vascular structures (arrow) are prominent within fat tissue. Mucosal cysts are seen incidentally in the left maxillary sinus. (**B**) Axial MRI short tau inversion recovery (STIR) image (TR 4700 ms; TE 75 ms; slice thickness 6 mm) of the same patient as in (**A**). High signal intensity is observed within the fat tissue (arrow). (**C**) Coronal MRI STIR image of the same patient as in (**A**). High signal intensity is observed within the fat tissue (arrow). (**D**) Axial soft tissue MDCT image of an underdeveloped pterygoid venous plexus. Vascular structures (arrow) appear sparse within the fat tissue. Mucosal cysts are seen incidentally in the left maxillary sinus. (**E**) Axial MRI STIR image of the same patient as in (**D**). No high signal intensity is observed within the fat tissue (arrow). (**F**) Coronal MRI STIR image of the same patient as in (**D**). No high signal intensity is observed within the fat tissue (arrow). Permission has been obtained to publish all figures.

In Le Fort I osteotomy, both soft tissue and bony structures must be assessed [31]. The pterygoid venous plexus is a key soft tissue structure [64] that can be visualized on CT in soft tissue mode by identifying vessels within fat spaces between the medial and lateral pterygoid muscles. The imaging appearance of the vessels within sparse fat suggests that the plexus is well developed. On short tau inversion recovery MRI, a well-developed pterygoid plexus is visible as an area of high signal in these regions (Figure 26B,C), whereas a poorly developed plexus shows a minimal signal (Figure 26E,F).

**Figure 27:**
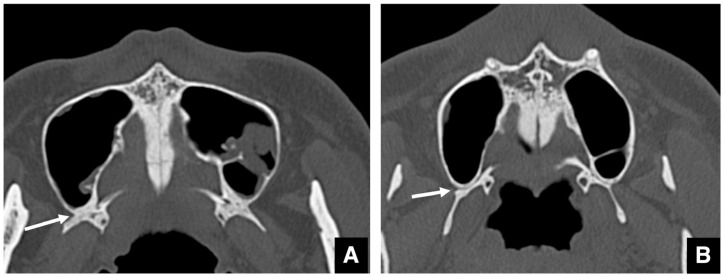
MDCT images (slice thickness, 3.0 mm) of the pterygomaxillary suture. (**A**) Axial bone window MDCT image shows a robust pterygomaxillary suture (arrow). Mucosal cysts are seen incidentally in the left maxillary sinus. (**B**) Axial bone window CT image shows a fragile pterygomaxillary suture (arrow). Permission has been obtained to publish all figures.

Table 4 lists the radiographic considerations regarding bony structures [65]. Proper separation of the pterygomaxillary suture is critical to avoid major complications during Le Fort I osteotomy [66,67,68]. CT in bone mode should be used to evaluate the fusion status of this suture. Narrow, heavily calcified sutures are difficult to divide (Figure 27A), whereas wide, poorly calcified sutures are easily separated (Figure 27B).

**Figure 28:**
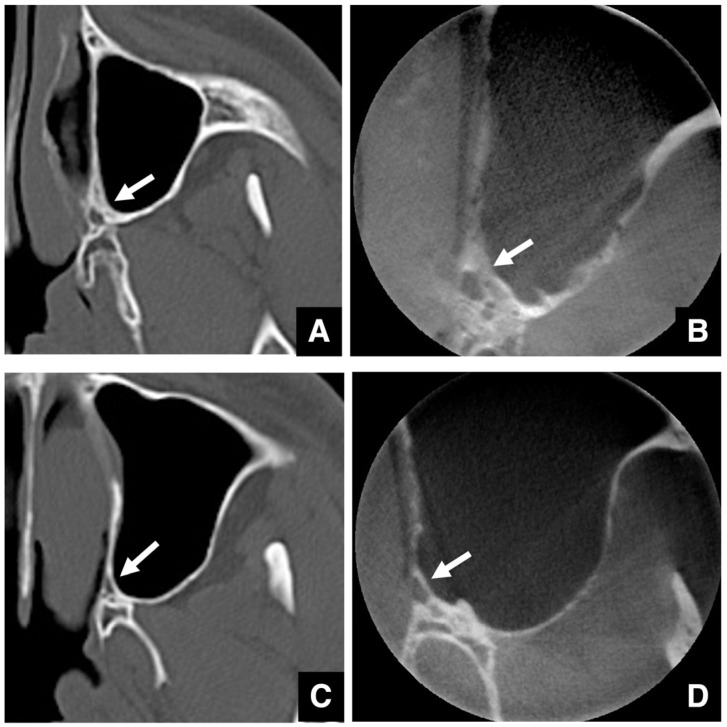
Axial images of the greater palatine canal obtained using multidetector computed tomography (MDCT; slice thickness, 3.0 mm) and dental cone-beam computed tomography (CBCT; slice thickness, 0.6 mm). (**A**) Axial bone window MDCT image of a patient with a thick-walled greater palatine canal (arrow). (**B**) Axial CBCT image of the same anatomical feature showing a thick-walled greater palatine canal with clearer delineation of the canal wall (arrow). (**C**) Axial bone window MDCT image of a patient with a thin-walled greater palatine canal (arrow). (**D**) Axial CBCT image of the same anatomical feature showing a thin-walled greater palatine canal with more clearly visualized canal margins compared with MDCT (arrow). Permission has been obtained to publish all figures.

Injury to the descending palatine artery, which runs through the greater palatine canal, can cause serious complications during suture separation [69,70,71]. The course and wall thickness of this artery must be assessed using CT in bone mode. Thick-walled canals carry a lower risk (Figure 28A), whereas thin-walled ones are more vulnerable (Figure 28B). Dental cone-beam computed tomography often provides clearer visualization of the palatine canal wall than conventional CT (Figure 28C).

**Figure 29:**
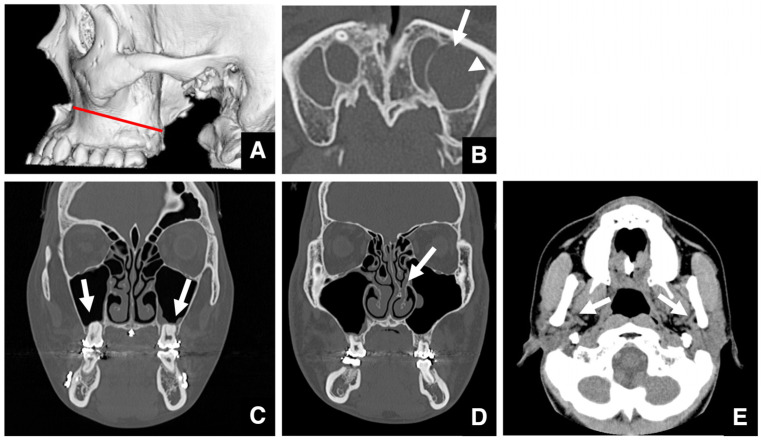
(**A**) MDCT images (slice thickness, 0.5 mm) illustrating anatomical considerations for Le Fort I osteotomy. A 3D image of the maxilla created from MDCT data. The shortest distance from the piriform rim to the greater palatine canal is measured (red line). (**B**) Axial bone window MDCT image (slice thickness, 3.0 mm) at the mid-maxillary sinus level. A well-developed maxillary sinus septum is observed (arrow). The sinus cavity is filled with soft tissue-like structures (arrowhead) typical of mucosal thickening and fluid retention. (**C**) Coronal bone window MDCT image at the molar level is used to evaluate the relationship between the floor of the maxillary sinus and the apices of the maxillary molars (arrows). (**D**) Coronal bone window MDCT image at the molar level. The nasal septum is deviated (arrow), but the size of the nasal cavity and the thickness of the lateral nasal wall appear normal. (**E**) Axial soft tissue MDCT image at the pterygoid level. Vascular structures (arrow) are prominent within fat tissue. Permission has been obtained to publish all figures.

The linear distance and bone thickness from the piriform aperture to the greater palatine foramen should be assessed on 3D CT (Figure 29A), in addition to the size, shape, and internal characteristics of the maxillary sinus. The presence of septations within the sinus varies individually and may increase the surgical complexity and the risk of postoperative inflammation (Figure 29B) [72].

To avoid root damage, the relationship between the maxillary sinus floor and the apices of the posterior teeth should also be assessed on panoramic and CT images (Figure 29C). CT can also reveal nasal septal deviation, nasal passage dimensions, and the thickness of the lateral nasal wall, which are relevant for surgical planning. Septal deviation may hinder postoperative nasal airflow, contributing to sinusitis. The width of the nasal passage and the thickness of the lateral wall affect the technical difficulty of osteotomy (Figure 29D). The course of the maxillary artery from the pterygopalatine fossa to the pterygopalatine fossa must also be evaluated in relation to the maxillary bone and the course of the maxillary artery (Figure 29E) [31]. Although the risk is extremely low, this helps to prevent massive bleeding or hematoma formation.

Careful evaluation of these factors enables jaw deformity surgery to be performed more safely and effectively.

### 2.10. Intraoperative and Immediate Postoperative Imaging Evaluation in Jaw Deformity Surgery

Table 5 outlines the imaging findings that must be evaluated during and immediately after orthognathic surgery for jaw deformities. These include vascular injury and massive hemorrhage, an abnormal condylar position or TMJ dislocation, nerve damage, accidental fractures, airway obstruction, and changes in pulmonary aeration (e.g., pneumothorax or atelectasis). However, unless a serious problem arises, it is extremely rare for it to be performed during surgery.

Although rare, unexpected fractures in areas not targeted during surgery can occur intraoperatively or shortly thereafter. Imaging plays a valuable role in identifying such fractures and confirming the proper reduction and fixation of bone segments.

Table 6 lists important radiographic considerations for imaging assessments performed after surgery. These include inflammatory changes, the bone healing status, abnormal fractures, the loosening or fracture of fixation plates, nerve injury, the development of temporomandibular joint disorders, PCR, temporomandibular joint displacement or dislocation, masticatory muscle condition during occlusion, and improvements in velopharyngeal insufficiency.

In Le Fort I osteotomy, bone healing is judged by the degree of calcification at the key junctions of the bilateral piriform rims, anterior maxillary walls, and infra-zygomatic crests. In cases of failed healing, imaging may reveal irregular bone resorption and clear separation between bone segments in these areas.

### 2.11. Studies Related to Pre- and Post-Treatment Evaluation of Jaw Deformities

**Figure 30:**
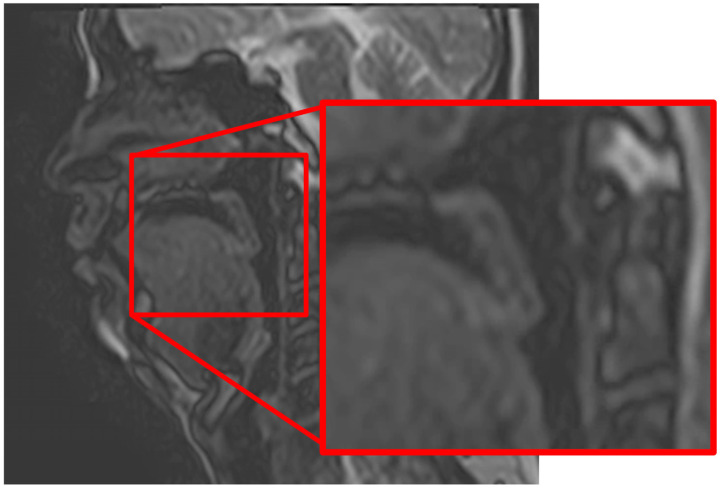
Cine-MR images (TR 3.2 ms, TE 1.6 ms, flip angle 45°, FOV 250 × 225 cm, slice thickness 8 mm, matrix 120 × 96) showing muscle movement and signal changes during articulation of “papa”. During articulation, the associated muscles move in coordination, accompanied by an increase in the signal intensity of each muscle. Supplementary Material Permission has been obtained to publish all figures.

**Figure 31:**
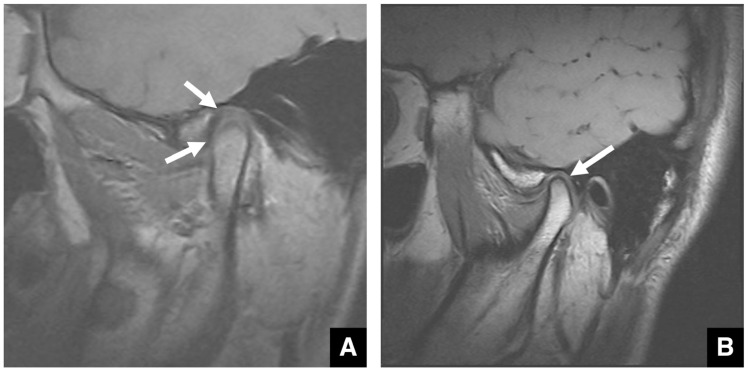
DCLS as an indicator of normal mandibular condyle development. (**A**) MR image (TR 1050 ms, TE 18 ms, slice thickness 3.0 mm) shows DCLS in the normal temporomandibular joint of a 10-year-old girl (arrows). (**B**) DCLS is not observed in an MR image of the normal temporomandibular joint in a 36-year-old man (arrow). Permission has been obtained to publish all figures.

Cine-MRI holds potential as a noninvasive method for the evaluation of speech and swallowing, making it a promising tool in assessing oral function before and after surgery for mandibular deformities (Figure 30, Supplementary Material) [73]. The presence of a double contour-like structure (DCLS) may serve as a marker of mandibular condyle growth and development, potentially acting as an indicator for the initiation of treatment in preoperative diagnosis (Figure 31) [74,75,76].

## 3. Conclusions

This review focuses on imaging-based diagnosis and pretreatment assessment in the management of jaw deformities. In current practice, cephalometric radiographs are supplemented with 3D reconstructions from CT data for morphological evaluation. Simulation tools are also used to predict outcomes, providing valuable information for treatment planning. However, the wide imaging field inherent in jaw deformity evaluation includes regions that may harbor other significant conditions. Therefore, we emphasize the importance of assessing not only the chief complaint but also the entire imaged area. The critical anatomical structures that must be evaluated with imaging for several representative surgical procedures (SSRO, Le Fort I osteotomy) are also described in detail. This review provides insights for immediate application in the clinical management of patients with jaw deformities. As the evidence-based literature does not cover all aspects of pretreatment evaluation, these criteria are based on the past experience of the authors.

## Figures and Tables

**Table 1:** Relationships between imaging modalities and the causes of malocclusion, skeletal growth, and the condition of the temporomandibular joint (TMJ). “S-level” indicates imaging modalities that are strongly recommended as essential examinations for diagnosis and treatment planning in jaw deformities, whereas “A-level” indicates auxiliary examinations that are recommended for specific purposes, such as the assessment of skeletal growth and development [1,2].

**Types of Malocclusion** **Tooth position abnormalities (high position, low position, displacement, transposition, rotation, inclination, crowding) → Panoramic radiography and CT (S-level)** **Angle classification (Class I, Class II Division 1, Division 2, Class III) → Cephalometric radiography and CT (S-level)** **Dental arch forms (narrow/V-shaped, saddle-shaped, spaced arch) → CT (S-level)** **Morphological abnormalities (alveolar, skeletal) → Cephalometric radiography and CT (S-level)** **Mesiodistal relationship abnormalities** **Mandibular mesial occlusion, mandibular distal occlusion, maxillary mesial occlusion, maxillary distal occlusion** **Horizontal relationship abnormalities** **Crossbite, scissor bite** **Vertical relationship abnormalities** **Open bite, edge-to-edge bite, deep bite** **Maxillary and mandibular protrusion** **Functional abnormalities → Cephalometric radiography, CT, and MRI (S-level)** **Causes of Malocclusion** **Systemic factors (genetics, congenital anomalies, metabolic disorders, endocrine disorders, nutritional deficiencies) → Panoramic radiography, Cephalometric radiography, Nuclear medicine, CT (S-level), and Carpal radiography (A-level)** **Local factors (oral habits, abnormalities in tooth number, shape, or eruption, soft tissue morphology abnormalities, dental and periodontal diseases, jawbone lesions, soft tissue lesions, etc.) → Panoramic radiography, Cephalometric radiography, Nuclear medicine, CT (S-level), and Carpal radiography (A-level)**

**Table 2:** Intraosseous observation checklists using images for sagittal split ramus osteotomy (SSRO).

**Setting the osteotomy line** **1. Evaluation of the position of the mandibular canal and surrounding bone quality** Presence or absence of mandibular canal bifurcation and course of resection site Distance from the buccal wall of the mandibular canal to the buccal cortical bone margin Bone quality around the mandibular canal (CT number) **2. Distance from the mandibular notch to the lingula** Whether it is 14 mm or more **3. Morphology of the mandible and variation in cortical bone thickness** Cortical bone thickness in the medial osteotomy area and lateral osteotomy area **Enhanced safety through improved visibility** **1. Medial curvature of the mandibular ramus** Mandibular ramus is straight or strong curvature **2. Course of small blood vessels along the bone surface** Depression of the buccal-lingual cortical bone from the mandibular fossa to the mandibular ramus region, trabecular bone defect **Improved success rates through enhanced postoperative bone integration** **1. Degree of interference between bone segments** The presence or absence of interference between the proximal and distal bone segments formed during virtual mandibular deformity surgery

**Table 3:** Observation checklists using images for genioplasty.

**Enhanced safety through improved visibility** **1. Course of the submental artery and its branches** Identification of the submandibular gland and its medial aspect **2. Course of the sublingual artery and the mental nerve** Identification of the mentalis muscle, hyoglossus muscle, and anterior belly of the digastric muscle and their medial aspects (Observed within the fatty tissue beneath the chin) **3. Course of small blood vessels along the bone surface** Degree of looping at the mentum foramen during opening, course of the incisive branch **Setting the osteotomy line** **1. Position of the mental foramen and course of the incisive branch** Record the position of each root for each lower tooth **2. Position of the mandibular anterior tooth roots** Record the thickness of the cortical bone using the mandibular teeth as a reference point **3. Variation in cortical bone thickness in the anterior mandible** Depression of the cortical bone in the mandibular anterior region, trabecular bone defect

**Table 4:** Observation checklists for the maxilla and surrounding bony structures using images for Le Fort I osteotomy.

**Improving safety through prediction of surgical difficulty and complication** **1. Circumstance of pterygoid venous plexus** Carefully observe the fat tissue surrounding the medial and lateral pterygoid muscles to assess the development of the internal vascular structures (using CT and MR to evaluate the size of the fat tissue) **2. Fusion status of the maxillary tuberosity and pterygomaxillary suture** When the fusion is narrow and the degree of calcification (CT number) is low or when the fusion is wide and high **3. Course of the descending palatine artery and thickness of surrounding bone** When the palatal canal wall is thick, the probability of damage during transection is low. When it is thin, the possibility of damage cannot be ruled out. **Setting the osteotomy line** **1. Distance from the piriform rim to the greater palatine canal** Long, short, cortical bone thickness, presence or absence of bone irregularities **2. Evaluation of the size, morphology, and internal features (mucosa, septa, etc.) of the maxillary sinus** Size, shape, presence or absence of septa, presence or absence of masses, presence or absence of mucosal thickening **3. Relationship between the floor of the maxillary sinus and the apices of the molars** Describe the maxillary sinus floor and the apex of each molar tooth **4. Presence of nasal septal deviation, size of the nasal passages, and thickness of the lateral nasal wall bone** Presence or absence of nasal septal deviation, Size of the nasal passage, Thickness of the cortical bone of the lateral wall **5. Course of the mandibular artery** The course from the pterygoid cleft to the pterygopalatine fossa in the posterior maxilla

**Table 5:** Radiographic considerations during and immediately after surgery.

**Vascular injury and massive bleeding** **Mandibular surgery: facial artery and vein, maxillary artery, inferior alveolar artery and vein, sublingual artery and vein, retromandibular vein, pterygoid venous plexus** **Maxillary surgery: pterygoid venous plexus, maxillary artery, descending palatine artery** **Instrument breakage and foreign body intrusion** **Abnormal position of the mandibular condyle and temporomandibular joint dislocation** **Nerve injury** **Accidental fractures** **Airway obstruction** **Pulmonary aeration status**

**Table 6:** Radiographic considerations in the postoperative period.

**Inflammation** **Status of bone healing** **Abnormal fractures** **Loosening or fracture of bone fixation plates** **Nerve injury** **Temporomandibular joint disorders and progressive condylar resorption** **Displacement or dislocation of the temporomandibular joint** **Condition of the muscles during occlusion** **Velopharyngeal insufficiency**

## Data Availability

Not applicable.

## Supplementary Materials

The following supporting information can be downloaded at https://www.mdpi.com/article/10.3390/diagnostics16020367/s1.

## Abbreviations

CBCT: cone-beam computed tomography; MDCT: multidetector row computed tomography; MRI: magnetic resonance imaging; QOL: quality of life; TMJ: temporomandibular joint; 3D: three-dimensional; SSRO: sagittal split ramus osteotomy; SSRO: sagittal split ramus osteotomy; SNA: angle formed between the S-N plane and the nasion of the maxillary alveolar base; SNB: angle formed between the S-N plane and the nasion of the mandibular alveolar base; SD: standard deviation; IVRO: intraoral vertical ramus osteotomy; Ar: intersection of the mandibular ramus and the lower margin of the occipital bone base; PNS: posterior nasal spine; ANS: anterior nasal spine; 2D: two-dimensional; PCR: progressive condylar resorption; STIR: short τ inversion recovery.
